# Predictors of Hypertension among Adult Female Population in Kpone-Katamanso District, Ghana

**DOI:** 10.1155/2019/1876060

**Published:** 2019-06-11

**Authors:** Kwabena Acheampong, Jackim M. Nyamari, Daniel Ganu, Stella Appiah, Xiongfeng Pan, Atipatsa Kaminga, Aizhong Liu

**Affiliations:** ^1^Department of Epidemiology and Health Statistics, Xiangya School of Public Health, Central South University, Changsha, Hunan 410078, China; ^2^Department of Public Health, Adventist University of Africa, Nairobi 00503, Kenya; ^3^Department of Nursing, Valley View University, Accra 00233, Ghana

## Abstract

*Background. *Hypertension is an independent risk factor of cardiovascular disease, which is one of the biggest health challenges today. The objective of this study was to estimate the prevalence of the problem and other factors related to hypertension among women who are 25 years and above.* Methods*. A community-based cross-sectional study was conducted from September and November 2017. A multistage cluster sampling technique was used to select the study participants. The data was collected using a structured questionnaire and physical measurements adapted from “WHO STEPwise approach to chronic disease risk factor surveillance (STEPS).” Data were examined using the SPSS program IBM version 20. Descriptive statistics, including proportions, frequencies, and cross-tabulations, were used to summarize the study variables. A binary logistic regression was fitted variable with a* p* value of < 0.7. The bivariable analyses were entered in the multivariable analysis to control the possible effect of confounders. Multivariable logistic regression analysis was used to identify factors associated with hypertension. The Adjusted Odds Ratio (AOR) with a 95 % Confidence Interval (CI) was computed to show the level of certainty. A* p* value of <0.05 was considered statistically significant.* Results*. The study indicated that the prevalence of hypertension (BP ≥ 140/90 mmHg) was 33.8% (95% CI 27.4-40.2) which increased with age. At the time of the study, women aged 45-64 years [AOR =2.19, (95% CI: 1.11-4.34, p<0.02)] and 65+ years [AOR =5.13, (95% CI: 2.20-11.99), p<0.001] were two to five times as likely as those with age of 25-44 years to be hypertensive. Women who had a higher body mass index (BMI) ≥30 kg/m^2^were two times as likely as those with normal weight to be hypertensive [AOR 2.38, (95% CI; 1.14-4.95, p<0.02)]. Women who did not consume fresh fruit daily were three times as likely as those women who consume fresh fruit daily to be hypertensive [AOR 3.17 (95% CI; 1.05-9.55, p<0.04)].* Conclusion*. Increasing age, obesity, and women who did not consume fresh fruits daily were associated with hypertension, indicating opportunities for health education and other prevention measures.

## 1. Introduction

Cardiovascular disease is the top cause of death in women in every major developed country and most emerging economies [1]. It involves the heart or blood vessels which affect about 1.1 billion people worldwide, maintains the top independent contributor to the global burden of disease, and accounts for 17.9 million deaths annually [2].

Hypertension is a risk factor for cardiovascular disease [3], and the complications of uncontrolled hypertension include stroke, heart failure, coronary heart diseases, peripheral vascular disease, retinal hemorrhage, visual impairment, and renal impairment [4–7]. Its early detection, prevention, prompt treatment, and control in sub-Saharan Africa (SSA) are suboptimal [8]. This is due to a combination of lack of resources and health-care systems, barriers to absolute compliance with prescribed medications or drugs, lack of effective preventive strategies at a population level, and lack of sustainable drug therapy.

The economic or fiscal impact for a number of years lost due to ill-health or early death and the need to divert scarce resources to tertiary care are substantial. The adult people with hypertension in 2000 were 972 million and were estimated to increase by 60% to a total of 1.56 billion in 2025 [9]. The rise in its prevalence will occur largely in developing countries [10].

Although the number of people with hypertension in developed countries was projected to increase by 24% from 333 million to 413 million, an increase of 80% was projected for developing countries from 639 million to 1·15 billion. This alarming increase in hypertension projected by a previous study [10] portrays a serious problem for SSA countries. Collectively, previous population-based studies have shown that hypertension in Ghana is a common problem and is reported to be as high as 19-48 % [11–14].

Nevertheless, some district in Ghana still lacks cross-sectional data on the prevalence of hypertension, its risk factors, and how this is distributed. The objective of the study was to estimate the prevalence of the problem and other factors related to hypertension among women who are 25 years and above in the district.

## 2. Materials and Methods

### 2.1. Study Design

A cross-sectional study was conducted to determine the factors of hypertension among the adult female population in Kpone-Katamanso District, located in the Greater Accra region of Ghana. Data collection started in September and ended in November 2017.

### 2.2. Sampling Size and Method

The sample size was calculated on the basis of a previous study which recorded the prevalence of hypertension in population as 19 % or higher [14, 15] and error of margin as 5% with 95% confidence interval. The minimum sample size required for 25406 female populations was calculated as n = 234 participants. Since the sampling procedure was multistage and taking into consideration the design effect, the sample size was further increased by 1.2 times and nonresponse rate by 7%. The total calculated sample size was 300 participants (derived by Epi-Info software version 7).

**Figure 1:**
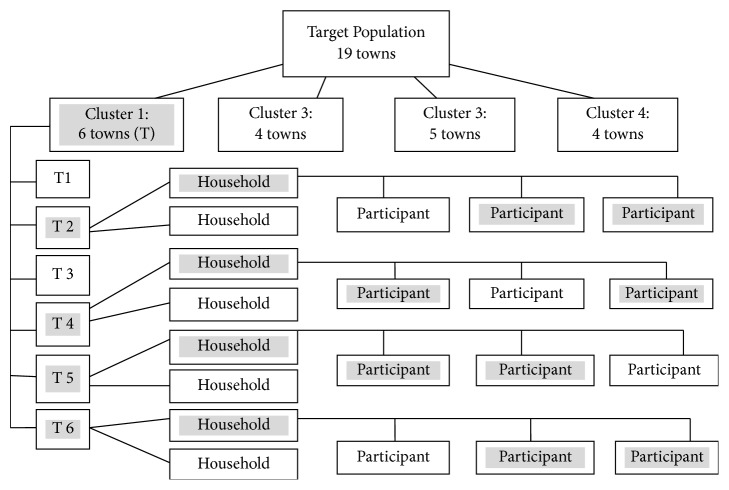
Flow chart of the sampling method. ^a^Town (T) or community represents a smaller cluster selected from the bigger cluster. ^b^A grey box shows that the box was selected to be sampled.

Multistage cluster sampling was employed for this study, which involved selecting communities and households. This study used a minimum number of the cluster by randomly choosing 4 communities from a list of 19 communities from the district. The basic sampling units in selected clusters were households. With a required sample size of 300 and one cluster(s) (representing four communities), the minimum sample required for each community was 37.5 households. Systematic sampling method was used to select the needed number of households in the cluster which applies to all the 4 selected communities. A total number of households in the district were 26,800 with an average household size of 4.1. However, the estimated number of households for the selected cluster was 5640. The interval for the cluster was then calculated by dividing the estimated number of households by the number of sample size (300) which gave an interval of 19th. A number within the sampling interval was randomly selected to get the first household to visit which was 4th. Consequently, households to visit were identified by adding up the sampling interval to the number selected. This was done until the 37.5 households required from each community making 150 households from a cluster were obtained. In households with more than two eligible persons, two were selected for the study using simple random sampling technique. In the nonappearance or absence of eligible persons in a selected household, at the time of the survey, the adjacent household was selected as shown in the above (Figure 1). A total of 216 adults consented and participated in the house survey, yielding a response rate of 72.0%.

### 2.3. Inclusion and Exclusion Criteria

Women aged 25 and above years in the selected study area who were permanent residents in the target area and who have been living there at least for six months were considered eligible for the study regardless of their status of blood pressure, while women who are unable to give response due to mental illness and those who were pregnant were excluded from the study.

### 2.4. Variables Measurement and Definition

Current Smoker was defined as a participant who reported smoking or any tobacco product within the last 30 days. Current Alcohol Drinker was defined as a participant who consumed alcohol within the last 30 days. Extra intake of salt was defined as participants who added a quantity of salt to food before consumption in addition to the usual amount added to food during cooking. Exercise is a planned physical activity that leads to visible improvements in health and general well-being. Hence physical exercise was defined as participants who engaged any vigorous or moderate exercise ranging from cardio exercises, running, brisk walking, skipping, weight lifting, and others, for a day or for most of the days in a week excluding manual workers like laborers, farmers, fishermen, and other manual workers. The fruit was characterized by daily intake of pawpaw, mango, grapes, watermelon, pears, strawberries, oranges, apples, pineapple, etc., and this was exclusively limited to fresh fruits.

#### 2.4.1. Dependent Variable

Women with systolic blood pressure ≥140mmHg and diastolic blood pressure ≥90mmHg or both were categorized as being hypertensive. The Joint National Committee 7 report released in 2003 categorizes blood pressure as follows: normal blood pressure (systolic (SBP)/diastolic (DBP) < 120/80 mmHg); prehypertension (SBP 120–139 mmHg or DBP 80–89 mmHg); stage 1 hypertension (SBP 140–159 mmHg or DBP 90–99 mmHg); and stage 2 hypertension (SBP ≥ 160 mmHg or DBP ≥ 100 mmHg) [16].

#### 2.4.2. Independent Variables

The sociodemographic characteristics are like age, marital status education status, employment status, and religion. Behavioral factors are like smoking, alcohol consumption, extra salt intake, daily fruit intake, fast and fried foods intake, and physical exercise and obesity.

### 2.5. Blood Pressure Measurements

The blood pressure measurements were taken using aneroid sphygmomanometer and stethoscope (pM-A01S). Period of rest before the BP was taken is variously given as “5 minutes” and “at least 5 to 10 minutes of rest.” The average of two systolic and diastolic blood pressure measurements was calculated and used as a dependent variable in the analysis. Hypertensive subjects were defined as those with systolic blood pressure (SBP) ≥140 mmHg and/or diastolic blood pressure (DBP) ≥90 mmHg or being previously diagnosed as hypertensive by any health professional.

### 2.6. Anthropometric Measurement

Weight was measured to the nearest 0.1kg using bathroom scale with shoes off. It was validated with standard pressure and corrected for zero inaccuracy. Height was measured without shoes to the closest 0.5cm using stadiometer. The BMI was computed as weight in kilograms divided by the square of the height in meters. BMI of participants were categorized as underweight (<18.5 kg/m^2^), normal weight (BMI≤18.5 to 24.9kg/m^2^), overweight (≥25 to 29.9kg/m^2^), and obese (BMI ≥30kg/m^2^) [17].

### 2.7. Data Collection and Management

Participants were interviewed using a structured questionnaire consisting of sociodemographic features, behavioral factors, and physical measurements adapted from “WHO STEPwise approach to chronic disease risk factor surveillance (STEPS).” The data collectors were four community health nurses supervised by two research officers. Training and practical demonstrations on the interview techniques and measurement procedures were given to data collectors for three consecutive days. Data reviewers were stationed at each town to ensure completeness of data recording by the field workers. Data collected was then entered into a safe computerized database using a unique code to ensure data accuracy.

### 2.8. Data Processing and Analysis

Data were coded and examined using the SPSS program IBM version 20. Descriptive statistics, including cross-tabulations, proportions, and frequencies, were used to recap the study variables. We performed an exploratory analysis using the Pearson Chi-square test (*χ*2). The association between hypertension and the independent variables was investigated by using the binary logistic regression and* p* values of <0.7 were considered as a variable selection criterion. Variables with a* p* value less than 0.7 were entered in the multivariable analysis to control the potential effect of confounders. Multivariable regression analysis was used to determine factors associated with the prevalence of hypertension. The Adjusted Odds Ratio (AOR) with a 95 % Confidence Interval (CI) was computed to show the level of certainty. A* p* value of <0.05 was considered as statistically significant.

### 2.9. Ethics Approval

Administrative permission was granted from the Kpone-Katamanso District Health Management Team. The participants were well informed about the study, its objectives, and method of data collection. Participants who agreed to be part of the survey gave their consent before being interviewed. The questionnaires were reviewed by the Valley View University and Adventist University of Africa Campus Institutional Research Ethics Review Committee.

## 3. Results

### 3.1. Background Characteristics of the Study Subjects

Two hundred and sixteen (with a response rate of 72.0%) participants were included in this study with the mean age of 49.6±13.3 years. Slightly more than two-thirds of the participants had no formal education: 100 (46.3%). More than one-third were not married and the majority of them were not employed: 84 (39.0%). The average weight of the study participants was 71.9±17.2kg, height was 160.4±5.5cm, and BMI was 27.9±6.3kg/m^2^. Out of 216 participants, 76 (35.2%) had normal weight, 4 (1.9%) were underweight, 70 (32.2%) were overweight, and 70 (32.2%) were obese (Table 1).

### 3.2. Prevalence of Hypertension

Blood pressure measurements were done to all the study subjects to check for hypertension. The mean systolic and diastolic blood pressure results were (Mean±SD) 125.7±18.4mmHg and 79.7±12.7mmHg, respectively. Out of 216 participants included, 73 (33.8%) of them were hypertensive, of which 26 (36.0%) are previously diagnosed as having the condition and 47 (64.0%) were newly diagnosed as hypertension.

### 3.3. Prevalence of Hypertension by Sociodemographic Characteristics

Prevalence hypertension was high among participants with age group of 65 years and above (57.0%) as compared to those with a low age group of 25-44 years (21.0%). The proportion of hypertension depicted an increasing trend with age. A statistically significant difference (p<0.001) was found between age and prevalence of hypertension. However, marital status education, employment, and religion were not statistically significant as summarized in Table 2.

### 3.4. Prevalence of Hypertension by Lifestyle-Related Factors

Prevalence of hypertension by lifestyle-related factors among study participants is summarized in Table 3. The prevalence of hypertension was higher among women who did not consume fresh fruits daily 68 (37%) than those who did 5 (17%) and the difference was significant (p<0.03). Although the proportion of hypertension was high among participants who reported to have added extra salt in their food after cooking, 56 (35.0%), as compared with those who did not, 17(31.0%), it was not statistically significant (p>0.60). A statistically significant difference (p<0.02) was found between BMI and the prevalence of hypertension.

### 3.5. Factors Associated with the Prevalence of Hypertension

Factors associated with the prevalence of hypertension among study participants are summarized in Table 4. Beyond the bivariate analysis of this study, the multivariable logistic regression analysis was computed. On bivariate analysis, a participant who consumes alcohol was about two times as likely as those who did not consume alcohol to be hypertensive and the crude odds ratio (COR) is 1.6 [95 % CI (0.87-2.98)]. Participants who did not have formal education were about two times as likely as those who have attained higher education to be hypertensive [COR 1.76 (95% CI; 0.53-5.86)].

The multivariate analysis showed the estimates of the association between independent variables and the prevalence of hypertension. After adjustment for age, marital status, education status, employment status, alcohol drinking, extra salt intake, fast foods intake, and body mass index, the participants who were 45-64 years [AOR =2.19, (95% CI: 1.11-4.34, p<0.02)] and 65+ years [AOR =5.13, (95% CI: 2.20-11.99), p<0.001] were two to five times as likely as those with age of 25-44 years to be hypertensive. Participants who were obese were two times as likely as those who have normal weight [AOR 2.38, (95% CI; 1.14-4.95, p<0.02)]. Participants who do not consume daily fresh fruit were about 3 times [AOR 3.17 (95% CI; 1.05-9.55, p<0.04)] as likely as those who consume daily fresh fruit to be hypertensive. No significant association was observed between the prevalence of hypertension and marital status or education status or employment status or alcohol drinking or extra salt intake or fast foods intake or physical exercise.

## 4. Discussion

The prevalence of hypertension (33.8% [95% CI 27.4-40.2]) established in this study corresponds to the findings of Addo et al. [11] and Burket [12], who reported that the prevalence of hypertension among women in Ghana was 30.2% and 30.7%, respectively. However, the prevalence found in our study is shown to be higher than the 25.5% reported for women in South West Nigeria rural communities [18] and the 15.0% reported for Eritrea national blood pressure survey [19], but lower than 48% prevalence reported among women in Accra by Hill et al. [13]. This change may be elucidated by differences in study location, sample size, and the average age of the study participants.

Age has been recognized as a nonmodifiable risk factor, attributing to an increased risk of cardiovascular problems, such as hypertension [20–23]. The present study is consistent with previous studies suggesting an association between increasing age and hypertension [24, 25]. One potential reason could be that vascular resistance increases with age when the vascular wall becomes harder. This change combined with age-linked conditions such as chronic heart failure which reduce cardiac output results in the increased incidence of hypertension among older age [26]. Therefore, increase of life expectancy among women in Ghana necessitates a practical and effective hypertension management strategy that targets its aging population.

Obesity and hypertension risks have been reported by previous studies [27–30], which support our finding. Similar results were observed in various studies done by Shihab et al. [31] and Tibazarwa et al. [32] which reported the occurrence of increasing body mass index and its significant impact on the development of hypertension. A study was done by Adedoyin et al. also reported a positive association with BMI and hypertension [33]. For that reason, the commencement of weight trimming program is required and would be more useful for young adults or middle-aged populations, to prevent the development of hypertension.

Our finding suggested that women who did not consume fresh fruit daily were found to be associated with the prevalence of hypertension. Borghi et al., Mirmiran et al., and Wu et al. provided evidence that fruit intake is associated with a lower risk of hypertension [34–36]. Other studies conducted among 28,082 female health professionals in the United States and a prospective cohort study among 745 residents aged 35 years without home hypertension at baseline from Ohasama, Japan, also reported a relationship between fruit consumption and decreased risk of hypertension [37, 38]. Lately, a longitudinal study conducted in China affirms that fruit intake was more robustly and significantly associated with lowering blood pressure than vegetable intake [39]. However, Nunez-Cordoba et al. reported no association between fruit intake and risk of hypertension [40], which needs to be researched further. The incidence of low daily fresh fruit intake and risk of hypertension in this study offers proof and support for the long-term education on the significance of fresh fruit intake and the prevention of hypertension.

Contrary to other studies, this study did not find any significant relationship between low education, unemployment, smoking, alcohol consumption, lack of physical exercise, and excessive salt intake which are significantly associated with hypertension in these studies [41–44].

## 5. Limitation of the Study

There are several limitations in this study. First, the study is cross-sectional which does not infer causal relationships. Besides, we took samples from only four towns and therefore caution should be taken to generalize the data for the entire population. Second, the determination of blood pressure was based on a single-day measurement instead of 24-hour ambulatory blood pressure, although two readings were taken. This strategy or approach might lead to misclassification of blood pressure category and introduce dilution bias, possibly underestimated or overestimated prevalence. Third, the proportion of the enrolled sample aged 65 years is higher than would be expected in the general population in the Greater Accra Region. This might lead to an oversampling of this age group. Fourth, the participation rate of this study was 72%, which is acceptable. However, the participation rate of 100% at recruitment is more confident and generalizable. Fifth, age of 65 years and above was assessed via open-ended strata, which may introduce some degree of residual confounding and selection bias. Again, despite comprehensive adjustment for multiple lifestyles and dietary factors, residual confounding by unmeasured or imprecisely measured hypertension risk factors may persist. Sixth, there was no census of the number of households in each town. However, secondary data from the 2010 Population and Housing Census was used, which might lead to underestimation of the true number of households.

## 6. Conclusion

The prevalence of hypertension was found to be high among women in the Kpone-Katamanso District. Older age, obesity, and women who did not consume fresh fruits daily were found to be significantly associated with hypertension. We recommend that for prevention of hypertension addressing no fruit intake and encouraging physical activity should be the primary option.

## Figures and Tables

**Table 1:** The background characteristics of the study subjects (n=216).

Variables	Frequency (%)
Age in years	
25-44	97(45.0)
45-64	82(38.0)
65+	37(17.0)
Marital Status	
No	69(32.0)
Yes	147(68.0)
Level of Education	
None	100(46.3)
Primary	52(24.1)
Secondary	48(22.2)
Tertiary	16(7.4)
Employment Status	
Employed	132(61.0)
Unemployed	84(39.0)
Religion	
Islam	6(3.0)
Christianity	210(97.0)
Blood Pressure	
Normal	126(58.3)
Prehypertension	17(7.9)
Stage 1 hypertension	59(27.3)
Stage 2 hypertension	14(6.5)
BMI (kg/m2)	
Underweight	4(1.9)
Normal	72(33.3)
Overweight	70(32.4)
Obese	70(32.4)

**Table 2:** Prevalence of hypertension by sociodemographic characteristics.

Variables	Normal (%)	Hypertensive (%)	Total (%)	P value
	(n=143)	(n=73)	(n=216)	
Age				<0.001
25-44	77(79.0)	20(21.0)	97(44.9)	
45-64	50(61.0)	32(39.0)	82(38.0)	
65+	16(43.0)	21(57.0)	37(17.1)	
Marital Status				0.256
No	42(61.0)	27(39.0)	69(31.9)	
Yes	101(69.0)	46(31.0)	147(68.1)	
Level of Education				0.685
No formal Education	63(63.0)	37(37.0)	100(46.3)	
Primary	34(65.0)	18(35.0)	52(24.1)	
Secondary	34(71.0)	14(29.0)	48(22.2)	
Tertiary	12(75.0)	4(25.0)	16(7.4)	
Employment Status				0.441
Employed	90(68.0)	42(32.0)	132(61.1)	
Unemployed	53(63.0)	31(37.0)	84(38.9)	
Religious Status				0.368
Islam	5(83)	1(17.0)	6(2.8)	
Christianity	138(66)	72(34.0)	210(97.2)	

**Table 3:** Prevalence of hypertension by lifestyle and comorbidities factors.

Variables	Normal (%)	Hypertension (%)	Total (%)	p value
	(n=143)	(n=73)	(n=216)	
Smoking Status				0.77
No	138(66.0)	71(97.3)	209(96.8)	
Yes	5(71.0)	2(29.0)	7(3.2)	
Alcohol status				0.13
No	108(69.0)	48(31.0)	156(72.2)	
Yes	35(58.0)	25(42.0)	60(27.8)	
Extra salt intake				0.60
No	38(69.0)	17(31.0)	55(25.5)	
Yes	105(65.0)	56(35)	161(74.5)	
Physical Exercise				0.83
No	98(67)	49(33)	147(68.1)	
Yes	45(65)	24(35)	69(31.9)	
Daily Fresh Fruit Intake				**<0.03**
No (Absence)	118(63)	68(37)	186(86.1)	
Yes	25(83.0)	5(17)	30(13.9)	
Fast & Fried Foods				0.12
No	93(63)	55(37)	148(68.5)	
Yes	50(74)	18(26.0)	68(31.5)	
BMI, kg/m^2^				**<0.02**
Underweight	4(100)	0(0.0)	4(1.9)	
Normal	55(71.0)	21(29.0)	72(33.3)	
Overweight	52(74)	18(26.0)	70(32.4)	
Obesity	36(51.0)	34(49)	70(32.4)	

**Table 4:** Binary and multivariable logistic regression analysis between predictor variables and hypertension among people living in Kpone-Katamanso District, Accra, Ghana.

Variables	Hypertension			
	*COR (95 % CI)*	*P value*	*AOR (95 % CI))*	*P value*
Age years		<0.001		
25-44	1	-	1	
45-64	2.46 [1.27-4.78]	**<0.010**	2.19 [1.11-4.34]	**<0.020**
65+	5.05[2.24-11.42]	**<0.001**	5.13[2.20-11.99]	**<0.001**
Marital status (Ref: Yes)	1	-	1	-
Single	1.41 [0.78-2.56]	0.257	1.42 [0.70-2.86]	0.322
Education status (Ref: Tertiary)	1	-	1	-
No formal Education	1.76 [0.53-5.86]	0.356	1.79[0.48-6.70]	0.389
Primary & Below	1.59 [0.45-5.64]	0.474	1.23 [0.31-4.92]	0.769
Secondary School	1.24 [0.34-4.49]	0.748	1.13 [0.28-4.60]	0.867
Employment status (Ref: No)	1	-	1	-
Unemployed	1.25[0.71-2.23]	0.441	1.59 [0.809-3.111]	0.179
Smoking (Ref: No)	1	-		
Current Smoking	0.78 [0.15-4.11]	0.767		
Alcohol (Ref: No)	1	-	1	-
Alcohol drinking	1.60[0.87-2.98]	0.129	1.55[0.766-3.143]	0.223
Extra Salt Intake (Ref: No)	1	-	1	-
Extra Salt Intake	1.19[0.62-2.30]	0.600	1.34 [0.635-2.808]	0.446
Daily Fresh Fruit Intake (Ref: Yes)	1	-	1	-
No Daily Fruit Intake	2.88[1.05-7.88]	**<0.039**	3.17 [1.05-9.55]	**<0.040**
Fast Foods Intake (Ref: no)	0.61[0.32-1.15]	0.125	0.69[0.33-1.45]	0.331
Physical Exercise (Ref: yes)	1	-		
No Physical Exercise	0.94[0.51-1.71]	0.834		
BMI, kg/m^2^				
BMI, kg/m^2^ (Ref: <25)	1	-	1	
BMI (≥25, <30) kg/m^2^	0.91 [0.44-1.89]	0.794	0.93[0.43-2.00]	0.840
BMI, (≥30) kg/m^2^	2.47 [1.24-4.92]	**<0.010**	2.38 [1.14-4.95]	**<0.020**

## Data Availability

The data used to support the findings of this study are available from the corresponding author upon request.
